# The Assessment of Diet Contaminated with Aflatoxin B_1_ in Juvenile Turbot (*Scophthalmus maximus*) and the Evaluation of the Efficacy of Mitigation of a Yeast Cell Wall Extract

**DOI:** 10.3390/toxins12090597

**Published:** 2020-09-15

**Authors:** Jinzhu Yang, Tiantian Wang, Gang Lin, Mingzhu Li, Ronghua Zhu, Alexandros Yiannikouris, Yanjiao Zhang, Kangsen Mai

**Affiliations:** 1 The Key Laboratory of Aquaculture Nutrition and Feed (Ministry of Agriculture), the Key Laboratory of Mariculture (Ministry of Education), Ocean University of China, Qingdao 266003, China; yangjinzhu@stu.ouc.edu.cn (J.Y.); tianttwang@163.com (T.W.); kmai@ouc.edu.cn (K.M.); 2 Institute of Quality Standards and Testing Technology for Agricultural Products, Chinese Academy of Agricultural Sciences, Beijing 100081, China; lingang@caas.cn; 3 College of Agriculture, Ludong University, Yantai 264025, China; ldulimingzhu@163.com; 4 Beijing Alltech Biological Products (China) Co., Ltd., Beijing 100600, China; jzhu@alltech.com; 5 Alltech Inc., Center for Animal Nutrigenomics and Applied Animal Nutrition, 3031 Catnip Hill Road, Nicholasville, KY 40356, USA; ayiannikouris@alltech.com

**Keywords:** Aflatoxin B_1_, adsorbent, physiological effects, intestinal microbiota, AFB_1_ residues, turbot, yeast cell wall extract

## Abstract

This study aimed to investigate the effects of dietary AFB_1_ on growth performance, health, intestinal microbiota communities and AFB_1_ tissue residues of turbot and evaluate the mitigation efficacy of yeast cell wall extract, Mycosorb^®^ (YCWE) toward AFB_1_ contaminated dietary treatments. Nine experimental diets were formulated: Diet 1 (control): AFB_1_ free; Diets 2–5 or Diets 6–9: 20 μg AFB_1_/kg diet or 500 μg AFB_1_/kg diet + 0%, 0.1%, 0.2%, or 0.4% YCWE, respectively). The results showed that Diet 6 significantly decreased the concentrations of TP, GLB, C3, C4, T-CHO, TG but increased the activities of AST, ALT in serum, decreased the expressions of CAT, SOD, GPx, CYP1A but increased the expressions of CYP3A, GST-**ζ**_1_, p53 in liver. Diet 6 increased the AFB_1_ residues in serum and muscle, altered the intestinal microbiota composition, decreased the bacterial community diversity and the abundance of some potential probiotics. However, Diet 8 and Diet 9 restored the immune response, relieved adverse effects in liver, lowered the AFB_1_ residues in turbot tissues, promoted intestinal microbiota diversity and lowered the abundance of potentially pathogens. In conclusion, YCWE supplementation decreased the health effects of AFB_1_ on turbot, restoring biomarkers closer to the mycotoxin-free control diet.

## 1. Introduction

Aquaculture is the fastest growing food production industry in the world, and by 2030 it is expected to provide 60 percent of the fish available for human consumption [1]. In aquaculture, plant-based protein alternatives are used to replace or partially replace fish meal since exclusive use of fish meal is not sustainable [2,3,4]. However, plant-based ingredients are easily contaminated with mycotoxins, which increase the health risks to fish [5,6]. Aflatoxin B_1_ (AFB_1_), the secondary metabolite of fungi *Aspergillus parasiticus* and *A. flavus* [7], is one of the most harmful mycotoxins [8]. In poultry and livestock, the detrimental effects of AFB_1_ include low productivity, high mortality of offspring, anorexia, poor growth, immune dysfunction, and AFB_1_ residues in edible animal parts [9,10,11,12,13,14,15]. In fish, there were studies of various species exposed to AFB_1_. Most of these studies concentrated on the impact on growth performance, liver lesions and immunosuppression induced by dietary AFB_1_. It had been reported that when sea bass (*Dicentrarchus labrax*) were fed a diet with 18 μg AFB_1_/kg of body weight, adverse effects (liver lesions and AFB_1_ residues in musculature) were induced [16]. Studies on rainbow trout (*Oncorhynchus mykiss*) indicated that toxic effects of AFB_1_ could be induced when fish were fed more than 0.05 μg AFB_1_/kg diet [17,18,19,20,21,22]. There was also plenty of research in nile tilapia (*Oreochromis niloticus*) [23,24,25,26], the poor growth performance was caused by more than 250 μg AFB_1_/kg in diet. Other relevant studies also included gibel carp (*Carassius auratus gibelio*) [27,28,29], grass carp (*Ctenopharyngodon idella*) [30], rohu (*Labeo rohita*) [31,32,33], red tilapia (*O. niloticus* × *O. mossambicus*) [34], channel catfish (*Ictalurus punctatus*) [35], tambaqui fingerlings (*Colossoma macropomum*) [36], beluga (*Huso huso*) [37] and Thai koi (*Anabas testudineus*) [38]. Usually the toxic effects of AFB_1_ occurred when the dose was greater than 100 μg/kg diet.

AFB_1_ is classified as Group 1 carcinogen of hepatocellular carcinoma to human by International Agency for Research on Cancer [39]. Consequently, food consumption of AFB_1_ presents a serious risk to human health [40,41,42]. Few studies showed the results of AFB_1_ residues in musculature in fish such as sea bass [16], gibel carp [27], tambaqui [36] and Thai koi [38], whereas other studies did not detect AFB_1_ tissue residues in nile tilapia [26] and red tilapia [34]. The disparities observed in these studies show the potential differences in responses and absorption/metabolization processes amongst fish species following AFB_1_ exposure. In addition, information on the effect of AFB_1_ on intestinal microbiota of fish is limited. Therefore, a more complete comprehensive understanding of the effects of AFB_1_ in fish is necessary.

AFB_1_ is stable and difficult to remove from contaminated feed; therefore, one of the strategies is to decrease its bioavailability [43]. Nowadays, adsorbing agents which could prevent AFB_1_ from being absorbed by the intestine are widely studied and used [41,43]. The studies of these products, including hydrated sodium calcium aluminosilicate, activated carbon, zeolites and yeast cell wall, etc., have been reviewed comprehensively [43,44,45,46,47,48]. Due to the negative effects of some of the inorganic adsorbents, such as adsorption of micronutrients [44,45], high inclusion rates [49] and limited adsorbing capacity to multiple mycotoxins [48,50], research has shifted to focus on composite-type mycotoxin adsorbents [43,51,52,53]. Yeast cell wall extract (YCWE) is an adsorbent that contains yeast cell wall, beer yeast powder, calcium carbonate and hydrated sodium calcium aluminosilicate, which have shown favorable effects in livestock and poultry challenged with mycotoxin exposure [54,55,56,57,58,59,60,61,62,63].

Turbot (*Scophthalmus maximus*) is an important commercial marine species in aquaculture. Recently, plant-based ingredients have been more widely used in the feeds of marine fish [2], with some inclusion levels higher than 50% of the total feed ingredients [64,65,66,67]. Research about the effects of AFB_1_ and mycotoxin adsorbents on turbot health and performance is lacking. Therefore, this study was aimed to investigate the impacts of AFB_1_ in turbot and evaluate the effects of YCWE on turbot fed AFB_1_ contaminated diets.

## 2. Results

### 2.1. Growth Performance

No significant difference was observed in FI and survival rate (*p* > 0.05) (Table 1). No significant difference was observed in WGR, SGR and FE in Diets 1–5 (*p* > 0.05) (Table 1). WGR and SGR of fish fed Diet 6 were lower than that of fish fed Diet 1 but no significant difference was observed (*p* > 0.05) (Table 1). The FE of fish fed Diet 6 was significantly lower than that of fish fed Diet 1 (*p* < 0.05) (Table 1). No significant difference was observed in moisture and crude protein content of fish (*p* > 0.05) (Table 2). No significant difference was observed in content of ash and crude lipid of fish fed Diets 1–5 (*p* > 0.05) (Table 2). The content of crude lipid of fish fed Diet 6 was significantly lower than that of fish fed Diet 1. The Diet 8 and Diet 9 resulted in significantly higher crude lipid content compared to fish fed Diet 6, but this was still significantly lower than that of fish fed Diet 1 (*p* < 0.05) (Table 2). Conversely, the content of ash of fish fed Diets 6, 7 and 8 was significantly higher than that of fish fed Diet 1 (*p* < 0.05) (Table 2). However, no significant difference of the ash content was observed between Diet 1 and Diet 9 (*p* > 0.05) (Table 2).

### 2.2. Biochemical Analysis of Serum

#### 2.2.1. TP, ALB and GLB

**Figure 1:**
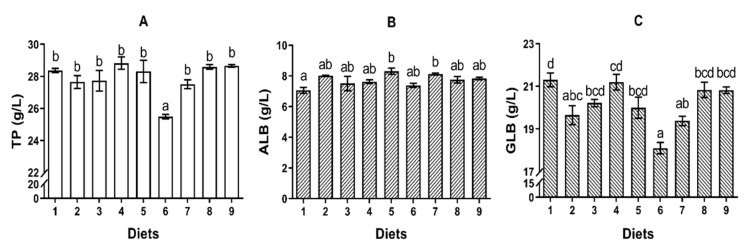
The effects of AFB_1_ and YCWE in serum TP, ALB and GLB concentrations of turbot. (**A**) Total protein (TP); (**B**) albumin (ALB); (**C**) globulin (GLB). Values represented are means ± S.E. of 3 replicate tanks. ^a, b, c, d^ Value bars not sharing a same superscript letter are significantly different (*p* < 0.05).

No significant difference was observed in the concentration of serum TP of fish fed Diets 1–5 (*p* > 0.05) (Figure 1A). The concentration of TP of fish fed Diet 6 was significantly lower than that of fish fed Diet 1 (*p* < 0.05) (Figure 1A). However, Diets 7, 8 and 9 resulted in significantly higher concentration of TP compared to Diet 6 (*p* < 0.05) (Figure 1A). No significant difference was observed in the concentration of serum ALB (*p* > 0.05) (Figure 1B). The concentration of GLB of fish fed Diet 2 and Diet 6 was significantly lower than that of in control diet (*p* < 0.05) (Figure 1C). However, Diet 8 and Diet 9 resulted in significantly higher concentration of GLB compared to Diet 6 (*p* < 0.05) (Figure 1C).

#### 2.2.2. IgM, C3, C4 and LZM

No significant difference was observed in the concentration of serum IgM and the activity of serum LZM among all groups (*p* > 0.05) (Table 3). No significant difference was observed on the concentrations of serum C3 and C4 of fish fed Diets 1–5 (*p* > 0.05) (Table 3). The concentrations of serum C3 and C4 of fish fed Diet 6 were significantly lower than that of fish fed Diet 1, while serum C3 and C4 concentrations of fish fed Diets 7, 8 and 9 were equal to those observed in fish fed Diet 1 (*p* > 0.05) (Table 3).

#### 2.2.3. TG and T-CHO

No significant difference was observed on the concentrations of serum TG and T-CHO of fish fed Diets 1–5 (*p* > 0.05) (Table 3). The concentrations of serum TG and T-CHO of fish fed Diet 6 and Diet 7 were significantly lower than that of fish fed Diet 1 (*p* < 0.05) (Table 3). In addition, no significant difference was observed on the concentrations of serum TG and T-CHO of fish fed Diet 8 and Diet 9 compared to Diet 1 (*p* > 0.05) (Table 3).

#### 2.2.4. ALP, AST and ALT

**Figure 2:**
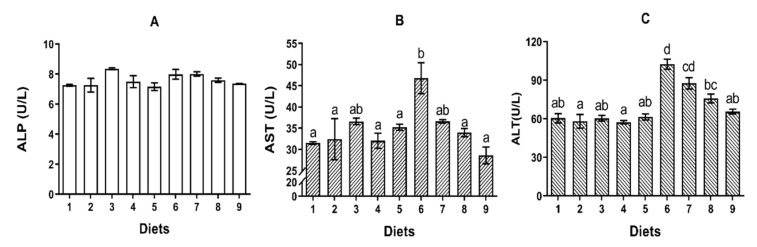
The effects of AFB_1_ and YCWE in serum ALP, AST and ALT activities of turbot. (**A**) Alkaline phosphatase (ALP); (**B**) aspartate aminotransferase (AST); (**C**) alanine aminotransferase (ALT). Values represented are means ± S.E. of 3 replicate tanks. ^a, b, c, d^ Value bars not sharing a same superscript letter are significantly different (*p* < 0.05).

No significant difference was observed in the activity of serum ALP among all groups (*p* > 0.05) (Figure 2A). No significant difference was observed in the activities of serum AST and ALT of fish fed Diets 1–5 (*p* > 0.05) (Figure 2B,C). The activities of AST and ALT in serum of fish fed Diet 6 were significantly higher than that of fish fed Diet 1 (*p* < 0.05) (Figure 2B,C). However, fish fed Diet 8 and Diet 9 had significantly lower activities of AST and ALT in serum compared to fish fed Diet 6 (*p* < 0.05) (Figure 2B,C).

### 2.3. Gene Expression in Liver

#### 2.3.1. Antioxidant Genes

**Figure 3:**
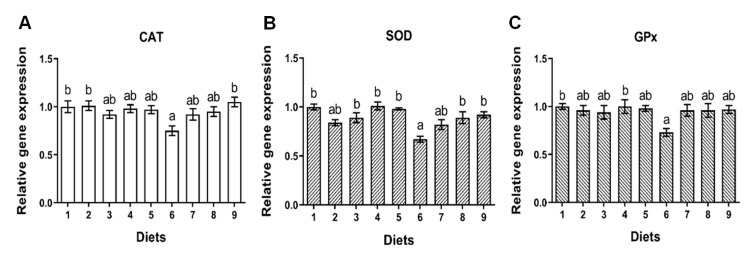
The effects of AFB_1_ and YCWE on antioxidant relative gene expressions in liver of turbot. (**A**) Catalase (CAT); (**B**) superoxide dismutase (SOD); (**C**) glutathione peroxidase (GPx). Values represented are means ± S.E. of 3 replicate tanks. ^a, b^ Value bars in a column not sharing a same superscript letter are significantly different (*p* < 0.05).

No significant difference was observed for gene expression of CAT, SOD or GPx of fish fed Diets 1–5 (*p* > 0.05) (Figure 3A–C). The gene expression of CAT, SOD and GPx of fish fed Diet 6 was significantly lower than that of fish fed Diet 1 (*p* < 0.05) (Figure 3A–C). Compared to Diet 6, the gene expression of CAT of fish was significantly heightened by Diet 9 (*p* < 0.05) (Figure 3A), and the gene expression of SOD of fish was significantly heightened by Diet 8 and Diet 9 (*p* < 0.05) (Figure 3B). No significant difference was observed for gene expression of CAT, SOD or GPx when fish were fed Diets 7, 8 and 9 compared to Diet 1 (*p* > 0.05) (Figure 3A–C).

#### 2.3.2. CYP1A, CYP3A, GST-ζ_1_

**Figure 4:**
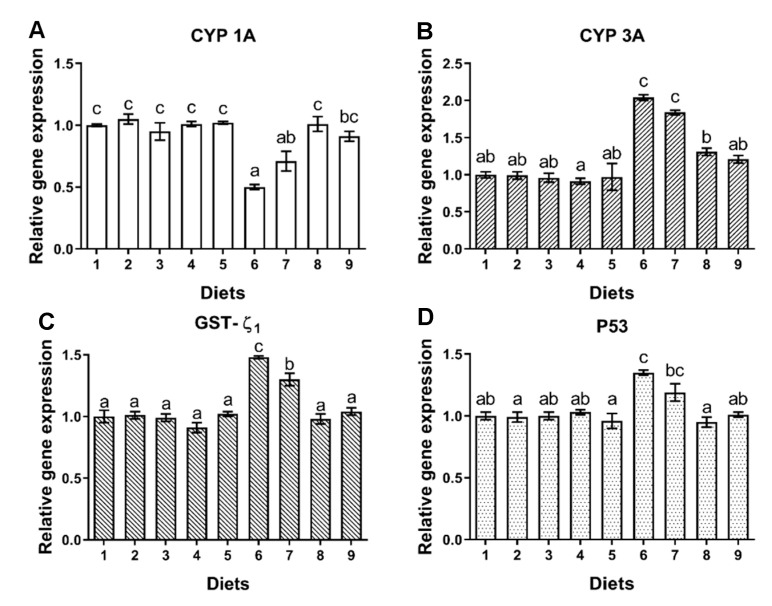
The effects of AFB_1_ and YCWE on CYP1A, CYP3A, GST-ζ_1_ and p53 expressions in liver of turbot. (**A**) Cytochrome p450 1A (CYP1A); (**B**) cytochrome p450 3A (CYP3A); (**C**) glutathione-S-transferase-zeta 1 (GST-ζ_1_); (**D**) p53: tumor suppressor protein p53. Values represented are means ± S.E. of 3 replicate tanks. ^a, b, c^ Value bars not sharing a same superscript letter are significantly different (*p* < 0.05).

No significant difference was observed on gene expression of CYP1A, CYP3A and GST-ζ_1_ of fish fed Diets 1–5 (*p* > 0.05) (Figure 4A–C). The gene expression of CYP1A of fish fed Diet 6 was significantly lower than that of fish fed Diet 1 (*p* < 0.05) (Figure 4A), while the expression was significantly higher in fish fed Diet 8 and Diet 9 (*p* < 0.05) (Figure 4A). The gene expression of CYP3A and GST-ζ_1_ of fish was significantly increased by Diet 6 (*p* < 0.05) (Figure 4B,C). Compared to Diet 6, the gene expression of CYP3A of fish was significantly lowered by Diet 8 and Diet 9 (*p* < 0.05) (Figure 4B), and the gene expression of GST-ζ_1_ of fish was significantly lowered by Diets 7, 8 and 9 (*p* < 0.05) (Figure 4C). No significant difference was observed for gene expression of CYP1A, CYP3A and GST-ζ_1_ when fish were fed Diet 8 and Diet 9 compared to Diet 1 (*p* > 0.05) (Figure 4A–C).

#### 2.3.3. Apoptosis Gene

No significant difference was observed on gene expression of p53 of fish fed Diets 1–5 (*p* > 0.05) (Figure 4D). The gene expression of p53 of fish fed Diet 6 was significantly higher than that of fish fed Diet 1 (*p* < 0.05) (Figure 4D). Compared to Diet 6, the gene expression of p53 of fish was significantly lowered by Diet 8 and Diet 9 (*p* < 0.05) (Figure 4D). No significant difference was observed for gene expression of p53 when fish were fed Diet 8 and Diet 9 compared to Diet 1 (*p* > 0.05) (Figure 4D).

### 2.4. Intestinal Microbiota

**Figure 5:**
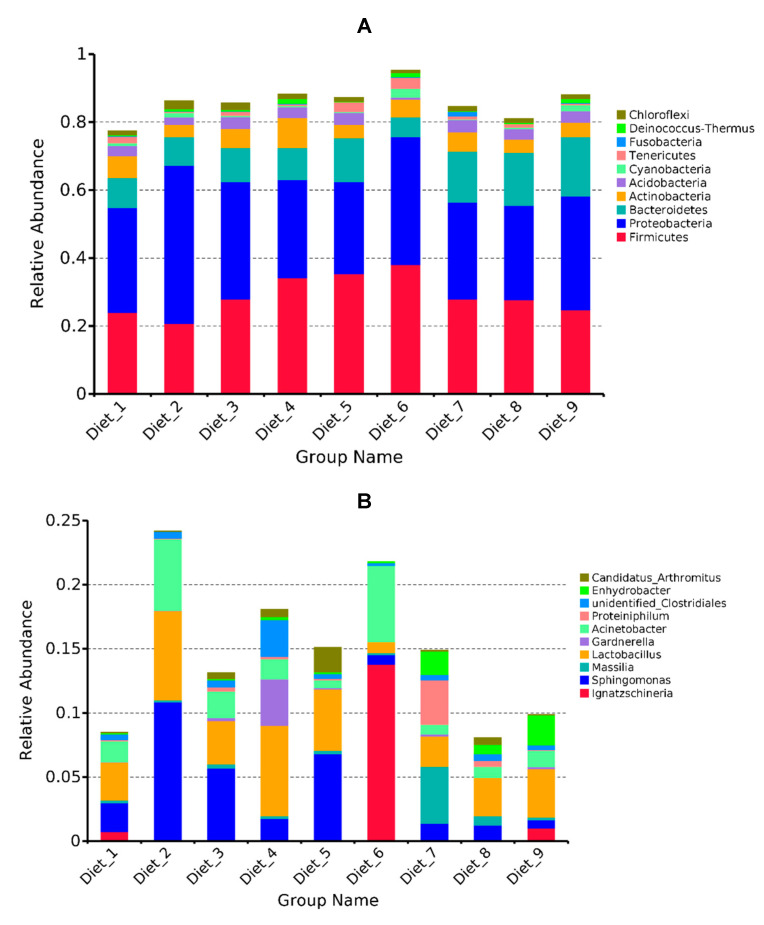
Taxonomy classification of reads at phylum (**A**) and genus (**B**) levels. Only top 10 most abundant (based on relative abundance) bacterial phyla and genera were shown in the figures.

After assembled, quality screened and trimmed, a total of 3,106,795 high quality valid reads were obtained, resulting in identification of 22,064 OTUs under 97% sequence similarity. These OTUs were assigned to 66 phyla, 82 classes, 185 orders, 375 families, and 1347 genera. Rarefaction curves, rank abundance and species accumulation boxplot showed that all samples reached the saturation phase, indicating adequate sequencing depth (Figure S1). At phylum level, Firmicutes, Proteobacteria, Bacteroidetes were the predominant bacterial phyla in turbot intestinal content across all groups, and Actinobacteria, Acidobacteria, Cyanobacteria, Tenericutes, Fusobacteria, Deinococcus-Thermus, Chloroflexi completed the top 10 most abundant phyla (Figure 5A). At the genus level, the top 10 most abundant genera were *Ignatzschineria*, *Sphingomonas*, *Massilia*, *Lactobacillus*, *Gardnerella*, *Acinetobacter*, *Proteiniphilum*, unidentified_Clostridiales, *Enhydrobacter*, Candidatus_*Arthromitus* (Figure 5B).

The alpha diversity indices indicated that no significant difference was observed on OTUs, Chao1 index, ACE index and Shannon index of fish fed Diets 1–5 (*p* > 0.05) (Table 4). OTUs, Chao1 index, ACE index and Shannon index were significantly lowered when fish were fed Diet 6 (*p* < 0.05) (Table 4). The Diet 2 and Diet 6 resulted in significantly lower PD whole tree index in turbot intestinal microbiota, especially, when fish were fed Diet 6, the PD whole tree index was even lower (*p* < 0.05) (Table 4). However, the Diets 7, 8 and 9 resulted in significantly higher alpha diversity indices including OTUs, Chao1 index, ACE index, Shannon index, and PD whole tree index (*p* < 0.05) (Table 4). No significant difference was observed on Simpson index among all groups (*p* > 0.05) (Table 4).

**Figure 6:**
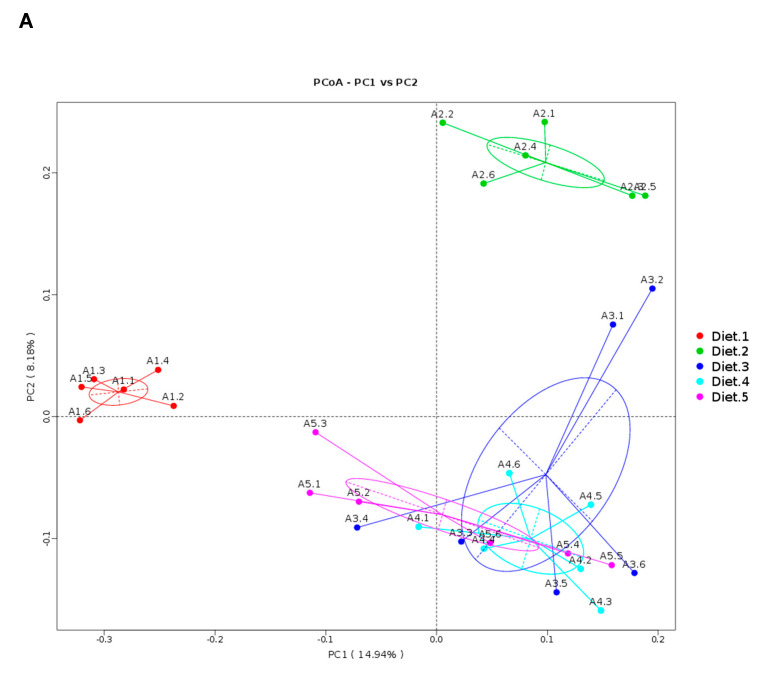

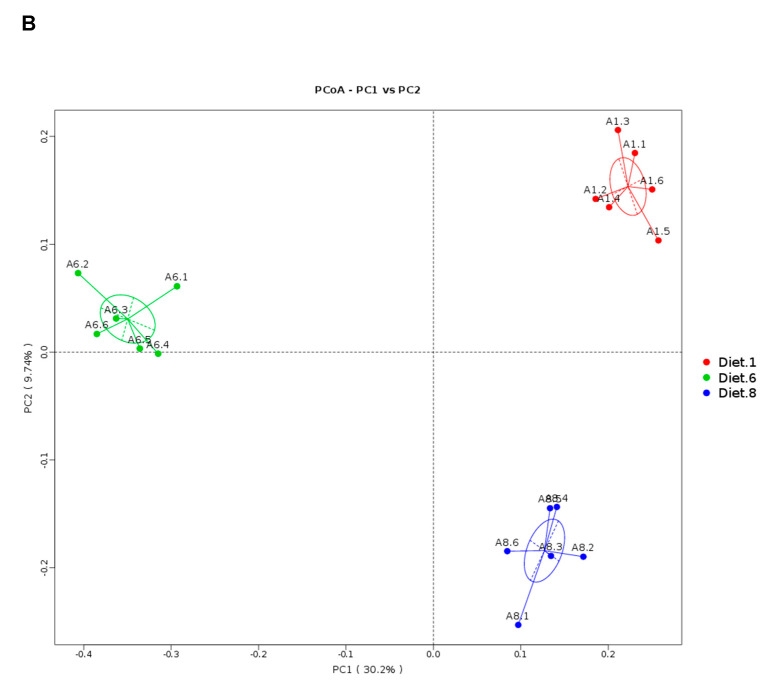
Principal Coordinate Analysis (PCoA) plot (**A**,**B**) in samples based on Unweighted Unifrac distances between Diet 1–5 or Diet 1, 6 and 8.

In Diets 1–5 groups, MRPP test confirmed the differences between groups were greater than differences within groups (Table S1). The results of Adonis test indicated the intestinal microbial community structure of fish fed Diet 1 or Diet 2 was significantly different from other groups, while the difference within Diets 3, 4 and 5 was not significant (Table S1). Similarly, the PCoA (Figure 6A) and UPGMA (Figure S2A) plot showed that samples clustered together according to the diets with a clear separation among Diet 1, Diet 2 and Diets 3–5. In Diet 1, Diet 6 and Diet 8 groups, MRPP test confirmed the differences between groups were greater than differences within groups (Table S2). The results of Adonis test indicated the intestinal microbial community structure in these three groups were significantly different from each other (Table S2). Similarly, the PCoA (Figure 6B) and UPGMA (Figure S2B) plot showed that samples clustered together according to the diets with a clear separation among Diet 1, Diet 6 and Diet 8.

**Figure 7:**
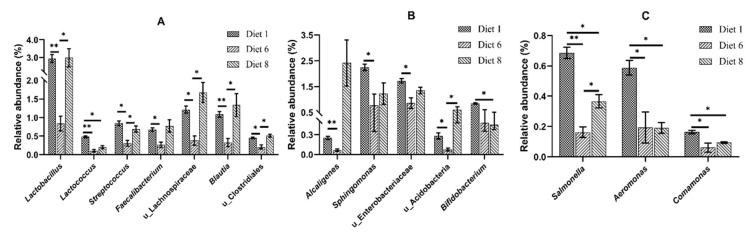
The MetaStat analysis of intestinal microbiota communities of juvenile turbot. (**A**–**C**) The significantly changed abundance at genus level in Diet 1, 6 and 8. (**A**) Genera belong to phylum Firmicutes. (**B**) Genera belong to phyla Proteobacteria, Acidobacteria, Actinobacteria. (**C**) Potential pathogenic genera belong to phyla Proteobacteria. u_ means unidentified. *: *p* < 0.05, **: *p* < 0.01.

MetaStat analysis was conducted to compare the relative abundance of intestinal bacteria at genus levels in Diet 1, Diet 6 and Diet 8 groups (Figure 7). The Diet 6 resulted in significantly lower abundance of genera *Lactobacillus*, *Lactococcus*, *Streptococcus*, *Faecalibacterium*, unidentified Lachnospiraceae, *Blautia*, unidentified Clostridiales, *Alcaligenes*, *Sphingomonas*, unidentified Enterobacteriaceae, and unidentified Acidobacteria, as well as potential pathogenic genera *Salmonella*, *Aeromonas* and *Comamonas* compared to Diet 1 (*p* < 0.05) (Figure 7). The Diet 8 resulted in significantly higher abundance of genera *Lactobacillus*, *Streptococcus*, unidentified Lachnospiraceae, *Blautia*, unidentified Clostridiales, unidentified Acidobacteria and *Salmonella* compared to Diet 6 (*p* < 0.05) (Figure 7). The Diet 8 resulted in significantly lower abundance of genera *Lactococcus*, *Bifidobacterium*, and potential pathogenic genera *Salmonella*, *Aeromonas* and *Comamonas* compared to Diet 1 (*p* < 0.05) (Figure 7).

### 2.5. AFB_1_ Residues in Serum and Muscle

**Figure 8:**
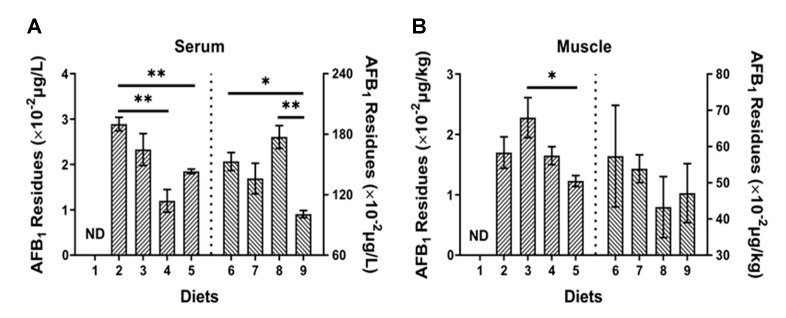
The effects of AFB_1_ and YCWE on AFB_1_ residues in serum and muscle turbot. (**A**) AFB_1_ residues in serum; (**B**) AFB_1_ residues in muscle. ND: Not Detected. Values represented are means ± S.E. of 3 replicate tanks. *: *p* < 0.05, **: *p* < 0.01.

No AFB_1_ residues in serum or muscle were detected in control group (Diet 1, Figure 8). The AFB_1_ residues in serum of fish fed Diet 4 and Diet 5 was significantly lower than that of fish fed Diet 2 (*p* < 0.05) (Figure 8A). The AFB_1_ residues in serum of fish fed Diet 9 was significantly lower than that of fish fed Diet 6 (*p* < 0.01) (Figure 8A). Besides, the AFB_1_ residues in muscle of fish fed Diet 5 was lower than that of fish fed Diet 2, fish fed Diet 8 and Diet 9 was lower than that of fish fed Diet 6, while no significant difference was observed (*p* > 0.05) (Figure 8B).

## 3. Discussion

In the present study, dietary AFB_1_ (20 and 500 μg/kg) did not remarkably affect the growth performance of turbot in a 67-day feeding trial. The adverse effects of higher level of AFB_1_ in diet on growth performance had been reported in several fish species. In nile tilapia, diets with AFB_1_ (2000 or 4000 μg/kg) remarkably reduced the weight gain (WG), FE, and the content of crude lipid [23], while results reported by Tuan et al. [25] and Deng et al. [26] demonstrated that WG, FI and FE were significantly reduced by 250 μg/kg or higher dietary AFB_1_. A similar result was also showed in tambaqui (500 μg/kg or higher level of AFB_1_) [36]. However, previous studies found that a diet with low levels of AFB_1_ could reduce the WGR, SGR and FI of grass carp (less than 147 μg/kg AFB_1_) [30]. Beluga fed diets with 75 or 100 μg/kg AFB_1_ affected WG and FE but not the SGR [37]. The effects of dietary AFB_1_ on the growth performance of fish is closely tied to the level of AFB_1_ in diet but is also dependent on the fish species, the development stage, the environment and the length of feeding terms.

It has been identified that dietary AFB_1_ could induce immunosuppressive response in aquatic animals, such as sea bass [16], grass carp [30], rohu [32], nile tilapia [68,69] and pacific white shrimp [70]. The level of TP and GLB can reflect protein synthesis capacity and immunity [32,71,72]. C3 and C4 are the key components of both classical and lectin pathways responsible for various immune effector functions [73]. In the current study, the concentrations of TP and GLB, as well as the concentrations of C3 and C4 in serum, were reduced by Diet 6, which suggested an immunosuppressive effect. It has been reported that the reduction of TP might be attributed to the hepatocellular damage [32], and reduction of GLB might be resulting from lymphocytolysis [74]. Generally, AST and ALT are recognized as biomarkers to identify the hepatic functions and cell membrane permeability. In the present study, turbot fed Diet 6 had higher activities of AST and ALT in serum indicating that AFB_1_ might cause hepatocellular damage in turbot. Similar results had been observed in other aquatic animals such as sea bass [16], nile tilapia [26], gibel carp [29] and pacific white shrimp [75]. The reduction of the concentrations of TG and T-CHO in serum induced by Diet 6 was similar when compared to previous studies performed in broiler chicks and ducks [76,77]. Due to the hepatocellular damage, the synthesis of TG and T-CHO was decreased, which was consistent with the lower concentrations of TP and GLB of serum as well. On the other hand, AFB_1_ requires metabolic activation by the cytochrome p450 enzymes system to generate AFB_1_-*exo*-8, 9-epoxide (AFBO), which can exert cytotoxic effects [78]. GSTs is one of the important detoxifiers of AFBO [79]. In the process of hepatocellular function, CYP1A and CYP3A play key roles in AFB_1_ activation [80,81,82], but CYP1A or CYP3A could also convert AFB_1_ to less toxic AFM_1_ or AFQ_1_, respectively [82]. In this study, the down-regulated expression of CYP1A and up-regulated expression of CYP3A and GST-ζ_1_ observed in fish fed Diet 6, might indicate that the affinity of CYP1A and CYP3A to AFB_1_ is different in hepatocytes of turbot. Moreover, AFBO, one of the oxidation products of AFB_1_ is easily bound to DNA, which could induce DNA damage [83,84]. Gene p53 could promote apoptosis when DNA damage is unrepaired [85]. Consequently, tissue antioxidant capacity may be compromised, and the oxidative metabolism of AFB_1_ may contribute further to oxidative stress [26,86], eventually leading to oxidative damage as well. This was consistent with the decreased expression of liver p53, CAT, SOD and GPx in group of Diet 6. A previous study in rohu also reported that both DNA and oxidative damage of liver were induced by dietary AFB_1_ [87,88].

In the present study, high-throughput sequencing was used to assess the overall intestinal microbiota community of juvenile turbot in response to dietary AFB_1_ and YCWE. The observation that the predominant phyla in the intestinal mucosa belonged to Firmicutes and Proteobacteria was in accordance with previous studies on turbot intestinal microbiota [89,90]. Compared with control group, turbot fed Diet 6 showed the lowest observed OTUs and phylogenetic diversity, and the microbiota community formed a different cluster from other groups. This was similar to a study in male Fischer 344 rat where AFB_1_ significantly decreased the observed OTUs and phylogenetic diversity [91]. In addition, Wang et al. reported the number of bacterial species at genus and phylum level were decreased by a dietary level of 5000 μg/kg AFB_1_ in pacific white shrimp [92]. The present study showed that Diet 6 significantly decreased the abundance of some potential beneficial microbiota, including *Lactobacillus*, *Lactococcus*, *Streptococcus*, *Faecalibacterium* genera, which are lactic acid producers [93]. It has been reported that lactic acid could efficiently degrade AFB_1_ into less toxic AFB_2_ and AFB_2a_ [94]. In addition, some studies had proved that some strains of *Lactobacillus*, *Lactococcus*, *Streptococcus* and *Bifidobacterium* could detoxify AFB_1_ by cell binding mechanisms [95,96,97]. Therefore, the intestinal bacteria might be involved in detoxification of AFB_1_. Besides, previous studies had reported the intestinal microbiota alteration of liver diseases’ patients. For example, in the host with cirrhosis and hepatic encephalopathy diseases, the decreased abundance of non-pathogenic bacteria Lachnospiraceae and Clostridiales were observed; in the host with non-alcoholic fatty liver disease, the decreased abundance of potential probiotics *Faecalibacterium* and *Bifidobacterium* were observed [98]. Therefore, the role of intestinal bacteria in liver disease induced by AFB_1_ is of great significance for further research.

Several studies have reported AFB_1_ residues in fish fed dietary AFB_1_. While few studies paid attention to the residues of AFB_1_ in serum, the current study was the first to study AFB_1_ residues both in serum and muscle of aquatic animals. The result showed that AFB_1_ residues in serum was higher than in muscle of turbot fed Diet 6. Furthermore, Han et al. reported AFB_1_ residues in muscle and ovaries of gibel carp [27], and similar findings had been observed in kidney and spleen of grass carp [30] and muscle, kidney and liver of tambaqui [36]. El-Sayed and Khalil reported AFB_1_ residues in musculature of sea bass at high level (about 5 μg/kg), the consumption of which could have negative effects on human health [16]. In contrast, two experiments with nile tilapia and red tilapia concluded that the consumption of fish muscle had no effects on human health as no AFB_1_ residue was detected when fish were exposed to AFB_1_ [26,34]. These differences might be the result of different uptake doses of AFB_1_ and different AFB_1_ metabolism pathways in different fishes.

In the present study, Diet 2 altered intestinal microbiota composition; however, there were no significant effects observed on growth performance, immune response and the diversity and abundance of intestinal microbiota in 67 days feeding trial. Nonetheless, low level of AFB_1_ residues in serum and muscle were observed in fish fed Diet 2. The AFB_1_ residues of fish muscle might accumulate more under long-term cultivation. Food contaminated with AFB_1_ residues may increase the risk to human hepatoma [99]. The safety level of AFB_1_ in human food has been set at 2 μg/kg by the European Union [100]; however, experts of FAO and WHO have given a guidance value for a provisional maximum tolerated daily intake of 1 ng AFB_1_/kg body weight per day [101]. Hence, even a low dose of AFB_1_ residues poses a danger to humans.

As a complex adsorbent, previous studies on YCWE focused on livestock and poultry. In the present study, YCWE also showed favorable mitigation efficacy to the adverse effects caused by dietary AFB_1_ in turbot. In addition, Diet 8 and Diet 9 lessened the immune function loss and liver damage induced by Diet 6. Apart from this, Diet 8 and Diet 9 resulted in lower AFB_1_ residues in serum and muscle. These results indicate the positive adsorbing capacity of YCWE to AFB_1_. Therefore, dietary YCWE could decrease the health risk resulting from feed consumption with AFB_1_ contamination. The results agree with previous studies in cows (10 g YCWE/cow per day) [58] and broilers (0.1% or 0.25% addition) [61,62,102,103]. Further, Diet 8 resulted in significantly higher alpha diversity and abundance of some potential beneficial bacteria compared to Diet 6; the abundance of potential pathogenic bacteria *Salmonella*, *Aeromonas* and *Comamonas* was lowered by Diet 8 compared to Diet 1. This derived a suggestion that YCWE might be beneficial to regulate intestinal microbiota communities.

## 4. Conclusions

The present study showed that 20 μg AFB_1_/kg diets did not affect the growth performance of turbot in a 67-day feeding trial. However, the AFB_1_ residues detected in serum and muscle of turbot suggested that the AFB_1_ intake at low level remains a potential health risk to human consumption of fish products. In addition, 500 μg AFB_1_/kg diet suppressed the immune response, induced liver damage (including reduced antioxidant capacity, decreased expression of antioxidant genes and increased expression of apoptosis genes in liver), increased AFB_1_ residues in serum and muscle, decreased the intestinal bacterial community diversity and reduced the abundance of some potential probiotics of turbot. However, the supplementation of 0.2% and 0.4% YCWE in 500 μg AFB_1_/kg diets resulted in liver function, immunity, AFB_1_ residues, intestinal microbiota communities and relative abundance of some potential probiotics more similar to that of the untreated control, which suggests that YCWE is an effective adsorbent to AFB_1_ in turbot feed.

## 5. Materials and Methods

### 5.1. AFB_1_ Preparation and YCWE Preparation

Reference Standard AFB_1_ was purchased from Pribolab (Qingdao, China) Technology Co., Ltd. The AFB_1_ was dissolved in absolute ethanol (AR, Sinopharm Chemical Reagent Co., Ltd., Shanghai, China) at 0.1 mg AFB_1_ per 1 mL ethanol.

Product of adsorbent (Mycosorb^®^) was provided by Beijing Alltech Biological Products (Beijing, China) Co., Ltd. YCWE was mixed with powder ingredients.

### 5.2. Experimental Diets

Based on previous studies establishing the negative effects of AFB_1_ on fish, 0 µg, 20 μg (Low contamination) or 500 μg (High contamination) of AFB_1_ per kg feed were included in the diets, resulting in the following nine isonitrogenous and isolipidic experimental diets: Diet 1 (control, basal diet): 0 µg AFB_1_/kg diet; Diets 2–5: 20 μg/kg AFB_1_ + 0%, 0.1%, 0.2%, or 0.4% YCWE; Diets 6–9: 500 μg/kg AFB_1_ + 0%, 0.1%, 0.2%, or 0.4% YCWE. The basal experimental diet was formulated as shown in Table 5. Fish meal, soybean meal and corn gluten meal were used as the main protein sources. Fish oil and soybean lecithin were used as lipid sources. Basal ingredients were purchased from Qingdao Great-seven Nutr-tech Co., Ltd (Qingdao, China). All the powder ingredients were thoroughly mixed, and then, the ethanol solution with AFB_1_ was re-dissolved in water and mixed with powder ingredients. After mixing all ingredients, the feed was pelleted with an approximate diameter of 3 mm and dried until constant weight at 55 °C in a ventilated oven. The practical content of AFB_1_ was detected by Beijing Alltech Biological Products (Beijing, China) Co., Ltd. The feeds were stored at −20 °C without light until the start of feeding. The chemical composition and AFB_1_ content in feeds were shown in Table 6.

### 5.3. Fish Husbandry and Sample Collection

Juvenile turbot (*Scophthalmus maximus* L.) was purchased from one commercial farm in Haiyang (Shandong, China). The feeding trial was carried out in Huanghai Aquaculture Co. Ltd. Prior to the start of the experiment, fish were acclimated to a commercial diet for two weeks with flowing water. Then the fish were fasted for 24 h and weighed (initial body weight of 12.43 ± 0.02 g). A total of 810 fish were randomly distributed to 27 cylindrical fiberglass tanks (200 L) in an indoor rearing system with flow-through seawater. In each tank 30 fish were cultured. The nine diets were randomly assigned to 27 tanks (three replications each group). Fish were fed to apparent satiation twice per day (08:00 and 18:00 h) for 67 days. The seawater of 2/3 volume was exchanged twice daily. To avoid the loss of AFB_1_, the experimental diets were stored in refrigerator (−20 °C), and a small portion of the feed was weighed to feed the fish every week. During the feeding trial, temperature was 12–14 °C; salinity was 30–33 ‰; pH was 7.5–8.0; dissolved oxygen was higher than 7 mg/L.

At the end of feeding trial, fish were fasted for 24 h, and then, all surviving fish were counted and weighed. After that, 2 fish of each tank were randomly selected and stored at −20 °C for whole-body analysis. Six fish of each tank were randomly selected to collect blood from caudal vein using 1 mL syringes. After clotting on ice, the serum was obtained by centrifugation with 3000 rpm for 10 min at 4 °C and stored at −80 °C for biochemical analysis. For enzyme activities and gene expressions analysis, 6 fish of each tank were randomly selected and dissected. The liver was obtained, transferred into 2 mL sterile tubes (Axygen, USA), frozen in liquid nitrogen and stored at −80 °C. For the analysis of intestinal microbiota, 2 fish of each tank were randomly selected. The exterior of fish was wiped with 70% ethanol, and the abdominal cavity was opened. After that, hind gut was obtained with sterile tools. The intestinal content was removed, the hind gut was transferred to 2 mL sterile tubes and immersed in liquid nitrogen immediately.

### 5.4. Growth Performance

Growth performance were calculated by using the following variables:

Weigh gain rate (WGR, %) = 100 × (finial body weight−initial body weight)/initial body weight

Specific growth rate (SGR, %/day) =100 × (Ln final body weight−Ln initial body weight)/days

Feed intake (FI, %/day) = 100 × total amount of feed consumptions/[(initial body weight + final body weight) ×2]/days

Feed efficiency (FE) = (final body weight−initial body weight)/total amount of feed consumptions

Survival rate (%) =100 × final number of fish/initial number of fish.

### 5.5. Feeds and Whole-Body Chemical Analysis

Chemical composition analysis of the feeds and the whole-body were performed following standard protocols of AOAC [104]: dry matter was measured by drying samples to a constant weight at 105 °C; crude protein was determined by measuring nitrogen (N × 6.25) using Kjeldahl method; crude lipid was determined by mineral ether extraction using Soxhlet method; ash content was determined by incineration of samples at 550 °C in a muffle furnace.

The moisture was calculated with following equation:

Moisture (%) = 100 × (W_1_ − W_2_)/W_1_;

W_1_: Wet weight of matter; W_2_: Dry weight of matter.

The ash content was calculated with following equation:

Ash (%) = 100 × W_3_/W_2_;

W_3_: Ash weight; W_2_: Dry weight of matter.

### 5.6. Biochemical Analysis of Serum

Hematological parameters were determined by using automated biochemistry analyzer (Roche/Hitachi cobas c 311 analyzer, Tokyo, Japan). GLB was calculated by subtracting ALB values from TP. The content of IgM, C3 and C4 and activity of LZM were determined by using commercial Fish ELISA kits and following manufacturer instructions (IgM: 17025, C3: 17181, C4: 17200, LZM: 17094, Quantikine^®^ ELISA kit, R and D Systems, Minnesota, MN, USA).

### 5.7. RNA Extract and Real-Time PCR

The total RNA of the liver was isolated using Trizol Reagent (9108; Takara Biotech, Dalian, China). Briefly, approximate 0.2 g liver tissue was homogenized in 1 mL RNAiso Plus using a tissue grinder. Then, chemicals were added in order following the reagent instruction. The RNA concentration and quality were assessed with NanoDrop ND-1000 Spectrophotometer (Thermo Scientific, Waltham, MA, USA). The integrity of extracted RNA was determined by electrophoresis on a 1.2% (*w*/*v*) agarose gel. After that, 1000 ng RNA was reverse transcribed to cDNA in 20 μL reactions using PrimeScript RT reagent Kit with gDNA Eraser (RR047A; Takara Biotech, Dalian, China). Then, real-time PCR was performed in a total 25 μL volume: 1 μL cDNA template (≤ 50 ng); 1 μL Forward primer (10 μM); 1 μL Reverse primer (10 μM); 9.5 μL DEPC-treated water (Sangon biotech, Shanghai, China); 12.5 μL TB Green™ Premix EX Taq II™ (RR820 A, Takara Biotech, Dalian, China). A two-step real-time PCR amplification program was used: 95 °C for 2 min and then 40 cycles of 95 °C for 10 s and 60 °C for 30 s. At last, melting curve analysis was used to ensure the specification of PCR product for each primer pair.

Specific primers for target genes and housekeeping genes, designed in NCBI, were synthesized by Sangon Biotech (Shanghai) Co., Ltd., and then the application efficiency was assessed (Table S3). All the real-time PCR analysis were performed using a quantitative thermal cycler (CFX96 Touch™ Real-Time PCR Detection System, Bio-Rad, Richmond, CA, USA). The genes expressions levels were normalized using relative quantitative method (2^−ΔΔCT^) referencing gene β-actin of turbot [105].

### 5.8. DNA Extract of Intestinal Microbiota and Sequencing Analysis

Genomic DNA sample was extracted from the intestinal mucosa layer using the QIAamp Fast DNA Stool Mini Kit (51604, Qiagen, Hilden, Germany) under sterile conditions (alcohol flame) following the manufacturer manual with some modifications [89,90]. PCR amplification of V4 region of 16S rRNA (515F/806R primer), quality and purity of PCR product were assessed by Beijing Novogene Genomics Technology Co. Ltd. (Beijing, China). Sequencing was conducted on an Illumina NovaSeq platform provided by Beijing Novogene Genomics Technology Co. Ltd. (Beijing, China).

For the sequence data analysis, Fast Length Adjustment of SHort reads (FLASH) was used to merge paired-end reads from the original DNA fragments when there were overlaps between reads1 and reads2 [106]. Sequencing reads were assigned to each sample with unique barcodes. Cutadapter was used to remove the adapter sequence, barcode sequence, primer sequence and to filter low-quality reads of raw reads [107]. The UCHIME algorithm was used to detect and remove chimeric sequences and obtain effective reads that would be used for further analysis [108]. After dereplication, abundance sort and discarding singletons reads, sequences with ≥97% similarity were clustered to the same OUTs (operational taxonomic units) using UPARSE [109]. Representative sequence for each OTU was screened for further annotation using Silva Database(v132) based on Ribosomal Database Project (RDP) classifier [110]. Alpha diversity indices (OTUs, Chao1, ACE, Shannon, Simpson and PD whole tree) were calculated with Quantitative Insights Into Microbial Ecology (QIIME) and displayed with R software (v 3.6.2) [111]. Beta diversity on unweighted UniFrac for Principal Coordinate Analysis (PCoA) and Unweighted Pair Group Method with Arithmetic mean (UPGMA) Clustering was calculated with QIIME and displayed with R software as well.

### 5.9. AFB_1_ Residues of Serum and Muscle

The AFB_1_ residues of serum and dorsal muscle of turbot were detected following the methods provided by Wang et al. [112]. This analysis was completed at the Institute of Quality Standard and Testing Technology for Agro-Products of CAAS (Beijing, China).

### 5.10. Statistical Analysis

Statistical software SPSS 22.0 for Windows (IBM SPSS corporation, Chicago, IL, USA) was used for the data analysis. Results were analyzed by one-way analysis of variance (ANOVA). Tukey’s multiple-range test was used for the multiple comparisons of group means. Differences were regarded as significant when *p* < 0.05. T-test was used for the comparisons of AFB_1_ residues in fish tissues of any two groups; “*” was marked when *p* < 0.05, and “**” was marked when *p* < 0.01.

Multi Response Permutation Procedure (MRPP) and Adonis test were employed to assess the difference of microbiota composition within or between groups using the vegan package in R software (v 3.6.2). MetaStat analysis [113] was conducted to identify the differential abundant taxa between groups.

### 5.11. Ethics Statement

The animal study was reviewed and approved by the Animal Care Committee of Ocean University of China (No. OUCFC2018001325, 24 August 2018).

## Figures and Tables

**Table 1:** Effects of aflatoxin B_1_ (AFB_1_) and yeast cell wall extract (YCWE) on growth performance and feed utilization in turbot *.

Diets	AFB_1_ (μg/kg)	YCWE (%)	IBW ^1^ (g)	FBW ^1^ (g)	FI (%/day)	WGR (%)	SGR (%/day)	FE	Survival (%)
1	0	0	12.46 ± 0.10	38.37 ± 0.27	1.03 ± 0.04	208.0 ± 4.1 ^ab^	1.68 ± 0.02 ^ab^	1.49 ± 0.06 ^c^	92.22 ± 1.11
2	20	0	12.36 ± 0.02	40.32 ± 2.64	1.15 ± 0.04	226.1 ± 21.1 ^b^	1.76 ± 0.10 ^b^	1.37 ± 0.02 ^abc^	96.67 ± 1.93
3	20	0.1	12.48 ± 0.03	38.78 ± 0.45	1.17 ± 0.04	210.7 ± 3.8 ^ab^	1.69 ± 0.02 ^ab^	1.31 ± 0.04 ^abc^	98.89 ± 1.11
4	20	0.2	12.40 ± 0.05	39.03 ± 2.17	1.12 ± 0.04	214.5 ± 16.1 ^ab^	1.71 ± 0.08 ^b^	1.38 ± 0.01 ^abc^	93.33 ± 0.00
5	20	0.4	12.41 ± 0.06	39.04 ± 0.50	1.08 ± 0.02	214.7 ± 2.6 ^ab^	1.71 ± 0.01 ^b^	1.43 ± 0.03 ^bc^	95.56 ± 1.11
6	500	0	12.45 ± 0.04	34.34 ± 0.15	1.10 ± 0.01	175.8 ± 1.9 ^ab^	1.51 ± 0.01 ^ab^	1.27 ± 0.01 ^ab^	95.55 ± 2.22
7	500	0.1	12.48 ± 0.06	32.79 ± 0.65	1.12 ± 0.01	162.6 ± 4.7 ^a^	1.44 ± 0.03 ^a^	1.20 ± 0.02 ^a^	92.70 ± 2.03
8	500	0.2	12.43 ± 0.06	34.26 ± 1.65	1.14 ± 0.04	175.6 ± 13.2 ^ab^	1.51 ± 0.07 ^ab^	1.22 ± 0.05 ^a^	97.78 ± 1.11
9	500	0.4	12.38 ± 0.06	34.08 ± 1.40	1.08 ± 0.03	175.1 ± 9.9 ^ab^	1.51 ± 0.05 ^ab^	1.29 ± 0.06 ^ab^	95.55 ± 2.22

* Values represented are means ± S.E. of 3 replicate tanks. ^a, b, c^ Values in a column not sharing a same superscript letter are significantly different (*p* < 0.05). ^1^ IBW: initial body weight; FBW: final body weight.

**Table 2:** Effects of AFB_1_ and YCWE on carcass composition in turbot *.

Diets	AFB_1_ (μg/kg)	YCWE (%)	Moisture (%)	Crude Protein ^1^ (%)	Crude Lipid ^1^ (%)	Ash ^1^ (%)
1	0	0	78.17 ± 0.24	64.21 ± 0.28	19.15 ± 0.31 ^c^	15.11 ± 0.06 ^a^
2	20	0	77.57 ± 0.29	64.41 ± 1.18	19.55 ± 0.21 ^c^	15.12 ± 0.31 ^a^
3	20	0.1	77.00 ± 1.41	65.86 ± 0.85	19.82 ± 0.01 ^c^	15.38 ± 0.17 ^a^
4	20	0.2	78.97 ± 0.34	66.54 ± 0.74	19.19 ± 0.10 ^c^	15.74 ± 0.04 ^ab^
5	20	0.4	79.22 ± 0.14	67.15 ± 1.15	19.18 ± 0.07 ^c^	15.73 ± 0.09 ^ab^
6	500	0	79.46 ± 1.10	66.69 ± 0.55	16.53 ± 0.18 ^a^	16.47 ± 0.03 ^c^
7	500	0.1	79.59 ± 0.23	66.32 ± 0.11	16.99 ± 0.06 ^a^	16.53 ± 0.06 ^c^
8	500	0.2	78.56 ± 0.51	65.11 ± 0.19	18.18 ± 0.03 ^b^	16.28 ± 0.02 ^bc^
9	500	0.4	78.96 ± 0.31	65.43 ± 0.25	18.15 ± 0.12 ^b^	15.73 ± 0.06 ^ab^

* Values represented are means ± S.E. of 3 replicate tanks. ^1^ Expressed as a percentage of dry matter. ^a, b, c^ Values in a column not sharing a same superscript letter are significantly different (*p* < 0.05).

**Table 3:** Effects of AFB_1_ and YCWE on hematological parameters in turbot *.

Diets	AFB_1_ (μg/kg)	YCWE (%)	IgM (g/L)	C3 (g/L)	C4 (g/L)	LZM (U/L)	TG (mmol/L)	T-CHO (mmol/L)
1	0	0	3.26 ± 0.01	5.67 ± 0.15 ^b^	0.976 ± 0.028 ^b^	0.360 ± 0.014	13.43 ± 0.38 ^bc^	4.04 ± 0.08 ^b^
2	20	0	3.20 ± 0.03	5.56 ± 0.09 ^b^	0.933 ± 0.021 ^b^	0.349 ± 0.007	13.28 ± 0.26 ^bc^	4.27 ± 0.21 ^b^
3	20	0.1	3.21 ± 0.03	5.53 ± 0.08 ^b^	0.967 ± 0.035 ^b^	0.366 ± 0.005	13.79 ± 0.12 ^c^	4.06 ± 0.03 ^b^
4	20	0.2	3.10 ± 0.07	5.38 ± 0.14 ^b^	0.912 ± 0.014 ^ab^	0.349 ± 0.011	14.07 ± 0.50 ^c^	4.24 ± 0.24 ^b^
5	20	0.4	3.09 ± 0.02	5.48 ± 0.12 ^b^	0.945 ± 0.029 ^b^	0.354 ± 0.006	13.82 ± 0.33 ^c^	3.85 ± 0.11 ^ab^
6	500	0	3.17 ± 0.04	4.47 ± 0.09 ^a^	0.809 ± 0.012 ^a^	0.347 ± 0.005	10.77 ± 0.12 ^a^	3.25 ± 0.19 ^a^
7	500	0.1	3.22 ± 0.02	5.28 ± 0.06 ^b^	0.892 ± 0.016 ^ab^	0.365 ± 0.007	10.57 ± 0.03 ^a^	3.22 ± 0.09 ^a^
8	500	0.2	3.19 ± 0.02	5.40 ± 0.06 ^b^	0.889 ± 0.015 ^ab^	0.353 ± 0.005	12.00 ± 0.20 ^ab^	3.68 ± 0.06 ^ab^
9	500	0.4	3.24 ± 0.06	5.46 ± 0.13 ^b^	0.930 ± 0.006^b^	0.363 ± 0.007	12.00 ± 0.45 ^ab^	3.70 ± 0.28 ^ab^

* IgM: immunoglobulin M; C3/C4: complement component C3/C4; LZM: lysozyme; TG: triglyceride; T-CHO: total cholesterol. Values represented are means ± S.E. of 3 replicate tanks. ^a, b, c^ Values in a column not sharing a same superscript letter are significantly different (*p* < 0.05).

**Table 4:** Richness and diversity indices of intestinal microbiota of experimental turbot *.

Diets	AFB_1_ (μg/kg)	YCWE (%)	Richness Estimates			Diversity Estimates		Phylogenetic Diversity
			OTUs	Chao1	ACE	Shannon	Simpson	PD whole tree
1	0	0	3918 ± 137 ^c^	4014 ± 147 ^c^	4203 ± 164 ^c^	9.21 ± 0.15 ^b^	0.973 ± 0.005	719.9 ± 24.9 ^d^
2	20	0	3219 ± 147 ^bc^	3306 ± 140 ^bc^	3467 ± 140 ^bc^	8.39 ± 0.49 ^ab^	0.983 ± 0.004	451.7 ± 23.8 ^bc^
3	20	0.1	3749 ± 188 ^c^	3831 ± 199 ^c^	3991 ± 222 ^c^	9.52 ± 0.16 ^b^	0.987 ± 0.003	464.0 ± 38.2 ^bc^
4	20	0.2	3439 ± 228 ^bc^	3551 ± 220 ^bc^	3738 ± 214 ^bc^	8.85 ± 0.42 ^ab^	0.972 ± 0.011	428.0 ± 29.8 ^b^
5	20	0.4	3402 ± 199 ^bc^	3496 ± 201 ^bc^	3656 ± 204 ^bc^	8.80 ± 0.48 ^ab^	0.971 ± 0.012	492.4 ± 41.1 ^bc^
6	500	0	1353 ± 161 ^a^	1411 ± 167 ^a^	1495 ± 176 ^a^	7.57 ± 0.35 ^a^	0.974 ± 0.010	211.8 ± 15.3 ^a^
7	500	0.1	3601 ± 173 ^c^	3715 ± 169 ^c^	3919 ± 174 ^c^	8.87 ± 0.36 ^ab^	0.971 ± 0.008	435.4 ± 26.1 ^b^
8	500	0.2	3499 ± 75 ^bc^	3569 ± 73 ^bc^	3714 ± 73 ^bc^	9.30 ± 0.17 ^b^	0.982 ± 0.005	579.8 ± 15.7 ^c^
9	500	0.4	2759 ± 237 ^b^	2821 ± 236 ^b^	2938 ± 244 ^b^	9.21 ± 0.15 ^b^	0.985 ± 0.002	396.4 ± 40.6 ^b^

* Values represented are means ± S.E. of 3 replicate tanks. ^a, b, c, d^ Values in a column not sharing a same superscript letter are significantly different (*p* < 0.05).

**Table 5:** Diet formulation of basal diet (% dry matter).

Ingredients	Content (%)
Fish meal	39.20
Soybean meal	15.68
Corn protein meal	8.00
Glutens	5.12
Beer yeast	2.50
Wheat flour	16.63
Taurine	1.00
L-Methionine	0.26
L-Threonine	0.18
L-Histidine	0.19
L-Lysine	0.74
Fish oil	8.00
Soya bean lecithin	1.00
Vitamin and mineral premix ^1^	1.00
Choline chloride	0.25
Ethoxyquin	0.05
Calcium propionate	0.10
Yttrium oxide	0.10

^1^ Vitamin and mineral premix (kg^−1^): retinyl acetate 675,000 IU; vitamin D_3_ 150,000 IU; DL-α-tocopherol acetate 6 g; vitamin K_3_ 1.2 g; thiamin nitrate 0.9 g; riboflavin, 1.35 g; pyridoxine hydrochloride 1.05 g; vitamin B_12_ 0.0075 g; D-calcium pantothenate 4.5 g; nicotinamide 6.75 g; folic acid 0.375 g; D-biotin, 0.015 g; L-ascorbyl-2-monophosphate-Na 15 g (based on L-ascorbic acid); inositol 10 g; Fe 20 g; Zn 9.6 g; Mn 5 g; Cu 0.6 g; Co 0.08 g; Se 0.04 g; I 0.08 g; moisture < 10%.

**Table 6:** Chemical composition and AFB_1_ concentrations of experimental diets.

Parameters	Diets								
	1	2	3	4	5	6	7	8	9
Chemical Analysis (% dry matter)									
Crude protein	52.8	52.5	52.8	53.8	53.1	52.4	53.3	53.1	53.8
Crude lipid	15.8	15.0	15.2	15.2	15.7	15.3	14.5	15.1	16.0
Ash	9.62	9.66	9.57	9.61	9.65	9.49	9.51	9.55	9.69
AFB_1_ Analysis (μg/kg)									
AFB_1_ (formulated value)	0	20	20	20	20	500	500	500	500
AFB_1_ (analyzed value)	ND ^1^	18	19	18	17	525	537	513	504
Adsorbents (%)									
YCWE ^2^	0	0	0.1	0.2	0.4	0	0.1	0.2	0.4

^1^ ND: Not Detected. ^2^ The content of wheat flour was partly replaced by the same addition of YCWE.

## Supplementary Materials

The following are available online at https://www.mdpi.com/2072-6651/12/9/597/s1. Figure S1: Rarefaction curves (**A**), Rank abundance (**B**) and Species accumulation boxplot (**C**) for all the intestinal microbiota samples. Figure S2: UPGMA clustering trees in samples (**A**,**B**) based on Unweighted Unifrac distances between Diet 1–5 or Diet 1, 6 and 8. Table S1: MRPP test and Adonis test of the Diet 1–5 groups’ microbial community structure of turbot. Table S2: MRPP test and Adonis test of the Diet 1, 6 and 8 groups’ microbial community structure of turbot. Table S3: Primer sequences, efficiency, amplicon size, annealing temperature and function for the genes profiled in real-time PCR.

## Abbreviations

AFB_1_	aflatoxin B_1_
AFBO	AFB1-exo-8, 9-epoxide
ALB	albumin
ALP	alkaline phosphatase
ALT	alanine aminotransferase
AST	aspartate aminotransferase
C3	complement component 3
C4	complement component 4
CAT	catalase
CYP1A	cytochrome p450 1A
CYP3A	cytochrome p450 3A
FE	feed efficiency
FI	feed intake
GLB	globulin
GPx	glutathione peroxidase
GSTs	glutathione-S-transferase
GST-ζ_1_	glutathione-S-transferase zeta-1
IgM	immunoglobulin M
LZM	lysozyme
p53	tumor suppressor protein p53
SGR	specific growth rate
SOD	superoxide dismutase
T-CHO	total cholesterol
TG	triglyceride
TP	total protein
WG	weight gain
WGR	weight gain rate
