# Supplementary material for: The Assessment of Diet Contaminated with Aflatoxin B1 in Juvenile Turbot (Scophthalmus maximus) and the Evaluation of the Efficacy of Mitigation of a Yeast Cell Wall Extract
Source: Toxins (Basel). 2020 Sep 15;12(9):597. doi: 10.3390/toxins12090597 (PMC7551837; doi:10.3390/toxins12090597)
Supplement: Supplementary file 1 [file toxins-12-00597-s001.pdf]

Supplementary Materials: The Assessment of Diet Contaminated with Aflatoxin B1 in Juvenile Turbot (*Scophthalmus maximus*) and the Evaluation of the Efficacy of Mitigation of a Yeast Cell Wall Extract

Jinzhu Yang, Tiantian Wang, Gang Lin, Mingzhu Li, Ronghua Zhu, Alexandros Yiannikouris, Yanjiao Zhang and Kangsen Mai

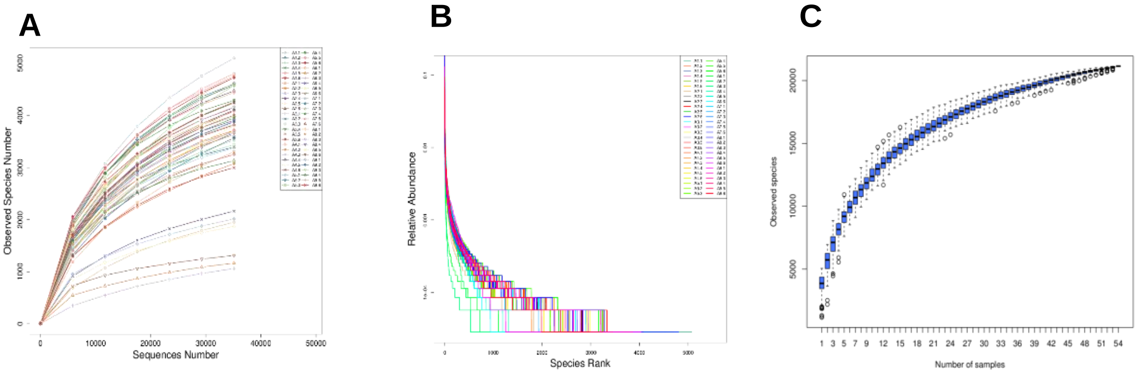

Figure S1. Rarefaction curves (**A**), Rank abundance (**B**) and Species accumulation boxplot (**C**) for all the intestinal microbiota samples.

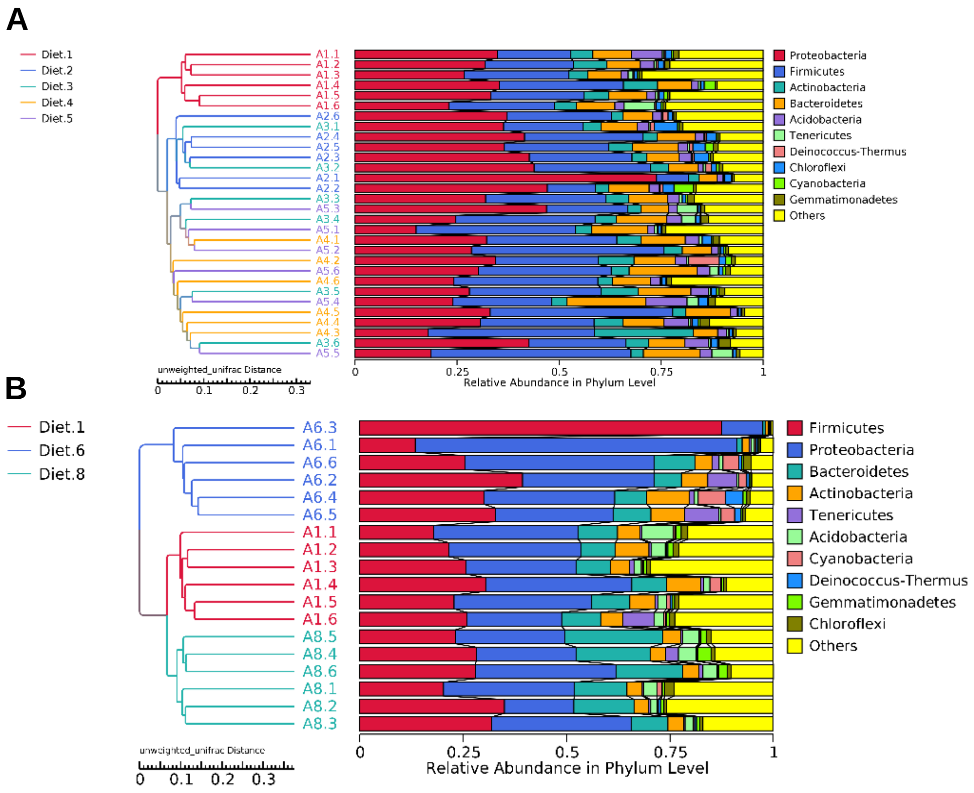

Figure S2. UPGMA clustering trees in samples (**A** and **B**) based on Unweighted Unifrac distances between Diet 1–5 or Diet 1, 6 and 8.

Table S1. MRPP test and Adonis test of the Diet 1–5 groups' microbial community structure of turbot. *.

Group	MRPP		Adonis
	A	Significance	*p*
Diet.1-Diet.2	0.187	0.001	0.002
Diet.1-Diet.3	0.062	0.002	0.003
Diet.1-Diet.4	0.068	0.002	0.002
Diet.1-Diet.5	0.078	0.001	0.007
Diet.2-Diet.3	0.123	0.002	0.002
Diet.2-Diet.4	0.152	0.005	0.006
Diet.2-Diet.5	0.124	0.004	0.003
Diet.3-Diet.4	0.004	0.317	0.547
Diet.3-Diet.5	0.015	0.082	0.219
Diet.4-Diet.5	0.019	0.040	0.282

* Values represent are means ± S.E. of 3 replicate tanks. A value greater than zero indicates that the difference between groups is greater than that within groups; significance value smaller than 0.05 indicates significant difference. Adonis test was calculated based on Unweighted Unifrac distances. *p*-value smaller than 0.05 indicates significant difference.

Table S2. MRPP test and Adonis test of the Diet 1, 6 and 8 groups' microbial community structure of turbot. *.

Group	MRPP		Adonis
	A	Significance	*p*
Diet.1-Diet.6	0.152	0.004	0.002
Diet.1-Diet.8	0.092	0.001	0.003
Diet.6-Diet.8	0.152	0.002	0.001

* Values represent are means ± S.E. of 3 replicate tanks. A value greater than zero indicates that the difference between groups is greater than that within groups; significance value smaller than 0.05 indicates significant difference. Adonis test was calculated based on Unweighted Unifrac distances. p-value smaller than 0.05 indicates significant difference.

Table S3. Primer sequences, efficiency, amplicon size, annealing temperature and function for the genes profiled in real-time PCR.

Gene	GenBank Accession no.	Primer Sequence (5'–3')		Amplicon Size (bp)	Annealing Temp. (°C)	Amplification Efficiency (%)
		Forward	Reverse			
CAT	MG253621.1	GGTTTGCCTGATGGCTTTCG	AAAAGGTCCAGGATGGGCAG	229	60	99
SOD	AWO99752.1	AAACAATCTGCCAAACCTCTG	CAGGAGAACAGTAAAGCATGG	165	60	99
GPx	AWP04239.1	GGACAAGTGGTGAAGCGGTA	TAAAGGTACGTGGGCAGGTC	74	60	100
p53	EU711045.1	CAAAGAGGCAGAGTGTGACC	TTGCACATGAAGCTCAGCAG	85	60	101
CYP1A	AJ310694.1	CAGCAAACCCTACCTGAGTC	CTTGCTTGATGAGAGCCTGT	131	60	103
CYP3A	JN216889.1	ATGAAGCCCTGATGCAGATG	CCTTGGGGATTATCACACCG	132	60	104
GST-z_1	DQ848966.1	GTCATTGCCTCTGGGATACAG	TCAAGAGCTGTGAAACCACG	113	60	100
β-actin	EU686692.1	CGTGCGTGACATCAAGGAG	AGGAAGGAAGGCTGGAAGAG	177	60	98

Gene full names: catalase (CAT); superoxide dismutase (SOD); glutathione peroxidase (GPx); tumor suppressor protein p53 (p53); cytochrome p450 1A (CYP1A); cytochrome p450 3A (CYP3A); glutathione-S-transferase zeta-1 (GST-ζ_1).
